# Remodelling and the fate of bone grafts in shoulder instability surgery

**DOI:** 10.1002/jeo2.70635

**Published:** 2026-01-22

**Authors:** Sarah Remedios, Jillian Karpyshyn, Ivan Wong

**Affiliations:** ^1^ School of Physiotherapy Faculty of Health, Dalhousie University Halifax Nova Scotia Canada; ^2^ Department of Orthopaedic Surgery Nova Scotia Health Halifax Nova Scotia Canada; ^3^ Division of Orthopaedic Surgery, Department of Surgery Dalhousie University Halifax Nova Scotia Canada

**Keywords:** anterior glenoid bone loss, arthroscopic anatomic glenoid reconstruction, distal tibia allograft, graft remodelling, graft resorption, shoulder instability, shoulder surgery

## Abstract

Anterior glenoid bone loss is a critical factor in the surgical management of recurrent shoulder instability. In cases where bone loss exceeds the threshold for soft‐tissue repair, bony augmentation is necessary to restore joint stability and prevent recurrence. However, there remains considerable debate regarding the optimal surgical technique and graft selection to maximise clinical outcomes and ensure long‐term graft survival. The Latarjet procedure is widely performed and associated with high success rates. Nevertheless, it is not without limitations, particularly the potential for postoperative complications and inconsistent graft resorption rates. Distal tibial allograft (DTA) has emerged as a biomechanically favourable alternative. Its osteochondral surface offers a strong anatomical match for the glenoid and has shown promising clinical results. Despite its growing adoption, critical questions remain regarding long‐term graft incorporation, structural remodelling, and recurrence rates. This review summarises the current evidence surrounding anterior glenoid bone augmentation techniques for shoulder instability, with a focus on graft selection, graft behaviour, and radiographic assessment. Special attention is given to the development and optimisation of DTA based on clinical outcomes and experience. Resorption patterns of the most common techniques and grafts employed, both auto‐ and allografts are discussed, and remodelling is defined. Understanding these remodelling patterns has practical implications for graft selection, fixation strategy, and postoperative management to optimise healing and long‐term stability.

**Level of Evidence:**

Level V.

**Abbreviations**
3D: three‐dimensional
AAGR: arthroscopic anatomic glenoid reconstruction
AP: anterior‐posterior
CT: computed tomography
DTA: distal tibial allograft
G: glenoid
GBL: glenoid bone loss
ICBG: iliac crest bone graft
ICGBT: iliac crest bone graft transfer
SI: superior‐inferior

## INTRODUCTION

In the setting of anterior shoulder instability with critical glenoid bone loss (GBL), the use of a bony augmentation procedure is crucial to prevent recurrence and revision surgery. ‘Critical′ GBL is variably defined, but a defect as low as 10% GBL can be considered for a bony augmentation procedure [25, 60, 74, 91]. Recurrence rates have been reported up to 75% when arthroscopic Bankart repairs are performed in the presence of GBL > 25%, leading to subsequent recurrent dislocations and further glenoid erosion [26, 46]. Multiple bone grafts have been proposed to address GBL and restore the contour and surface area of the glenoid, providing mechanical stability and preventing recurrent dislocations.

Bone grafting procedures date back to the early 1900s and have continued to develop since then [28, 45]. These procedures vary in technique and can be open or arthroscopic, and with an auto‐ or allograft. Autografts for shoulder instability commonly include a coracoid transfer in the Bristow and Latarjet procedures [54], as well as iliac crest bone graft transfer (ICBGT) [79], distal clavicle autograft [82], and scapular spine [71]. Allografts include the iliac crest allograft [51], and distal tibial allograft (DTA) [69, 88], which has become an increasingly popular technique for addressing recurrent anterior shoulder instability with GBL. These auto‐ and allograft techniques make up key surgical procedures to re‐establish shoulder stability by recreating the glenoid track following traumatic and/or recurrent instability [16].

During surgery, the bone grafts are placed against the native glenoid, in continuity with the articular surface, and over time the graft undergoes biological *remodelling*, during which *resorption* can occur, defined as the extent the added bone integrates into the natural bone over time [33, 34, 72]. Resorption typically stabilises once the graft maintains a normal‐appearing cortical outline in both axial and en‐face views where *healing* has occurred and *remodelling* has plateaued [22]. At this final stage, the graft′s structure supports long‐term joint stability and function.

As the use of auto‐ and allografts have become more popularly studied, concerns are highlighted regarding graft osteolysis or resorption [23, 87]. For example, Delgado et al [23] studied four bone augmentation techniques and reported graft osteolysis in 100% of the iliac crest allograft group, 50% in the iliac crest autograft group, 20% following open Latarjet, and 0% following arthroscopic Latarjet. High resorption can have possible surgical and clinical implications, such as prominent hardware leading to pain, stiffness, or instability recurrence [75]. Understanding how different grafts integrate into the anterior glenoid rim in the setting of a bony augmentation procedure is essential to optimise patient outcomes, reduce graft failure, and guide surgical decision making. This paper aims to evaluate resorption characteristics and define remodelling in the setting of bone grafting in shoulder instability using both autografts (i.e., iliac crest and coracoid process) and allografts (i.e., iliac crest and DTA).

### Defining and measuring glenoid bone loss

**Figure 1:**
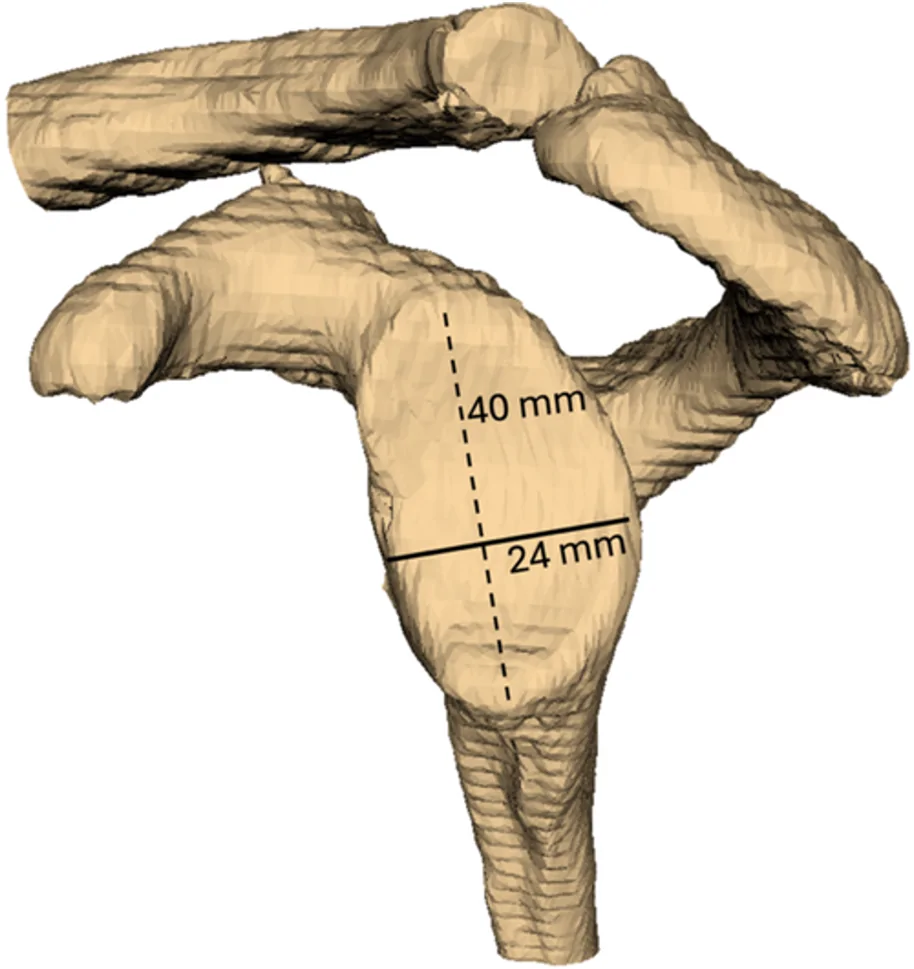
Measurement of glenoid bone loss (GBL) using 3D reconstruction and *en‐face* view demonstrating superior‐inferior (SI, 40 mm) and anterior‐posterior (AP, 24 mm) measurement. Estimated AP glenoid = [0.70 * (40 mm) + 2.53] = 30.53 mm.

Anterior GBL is one of the most important risk factors for recurrent dislocation; therefore, accurate GBL measurement is crucial in guiding treatment decisions. Advanced imaging with computed tomography (CT) is essential, with three‐dimensional (3D) reconstruction being preferred [42]. Multiple validated techniques have been described to quantify the amount of GBL [43, 53, 66, 70], however inconsistency exists in the literature for reporting GBL due to methodological variation [52, 84, 86]. Low inter‐rater reliability is particularly problematic when measuring GBL by drawing a circle of ‘best‐fit’, where size and placement of this circle on the correct area of cortical bone can be difficult [55]. To develop a more reproducible method for measuring GBL, our group investigated normal glenoid morphology and found a direct relationship between the superior‐inferior distance of the glenoid and the anterior‐posterior glenoid width [70]. By utilising the superior‐inferior and anterior‐posterior measures from the *en‐face* view of the 3D reconstructed CT, we can reproducibly determine the amount of glenoid missing using the formula: 0.70*H + 2.53; where *H* = superior‐inferior glenoid height [41, 70] (Figure 1). From this estimation, we can use a surface area calculation to determine amount of bone missing [8, 67].

### Graft options and surgical techniques

#### Autografts

##### Coracoid autograft

The Latarjet procedure is one of the most common autograft‐based procedures and historically has been considered the gold standard for treatment of recurrent instability with GBL. The coracoid process is harvested and transferred to the anterior rim of the glenoid to restore the bony architecture, as well as providing a ‘sling effect’ of the conjoint tendon, restoring shoulder stability [32, 54]. The Latarjet procedure was initially described with the use of two screws; however, recently the use of suspensory fixation has been advocated as an alternative technique [13]. Long term follow‐up of the Latarjet show low rates of recurrent instability, especially compared to the soft‐tissue stabilisation procedures [4, 6, 7, 44, 47, 77], but with variable complication reporting in both arthroscopic and open approaches [47]. Reported complications of the open Latarjet range from 7% to 30% [2, 21, 39], with arthroscopic Latarjet procedures having a similar rate (8.4%–23.4%) [18, 27, 44, 58]. While studies vary on what complications are considered, commonly reported complications include screw mispositioning, nerve injury, non‐union, and resorption [40].

##### Iliac crest bone autograft

The use of an iliac crest bone autograft transfer (ICBGT) has been previously described utilising a bicortical autograft attached via suspensory fixation methods or screws [5, 79]. The ICBGT is a free bone alternative that achieves anatomic glenoid reconstruction without the use of the sling effect. ICBGT has shown comparable outcomes to the Latarjet procedure, including clinical outcomes, frequency of recurrent dislocations, and complication rates [10, 62]. Recurrent instability and complications are reportedly low (<5%), which includes the rate of hardware complications [10, 56].

##### Distal clavicle autograft

The use of a distal clavicle autograft has been described previously for its combination of being readily available (i.e., harvested from the patient) and osteochondral properties [81]. Tokish in 2014 describes it to compare favourably with the coracoid for its size (‘thickness and bulk’).

##### Scapular spine autograft

The scapular spine autograft is another option but similar to the distal clavicle autograft is not commonly used. The differentiating advantage of the scapular spine autograft is the donor site is in the same area of arthroscopic portals thereby reducing multisite morbidity [90]. Moroder et al. presented a technical note describing a cerclage technique using a tricortical scapular spine autograft [61]. There is limited research on patient outcomes, but one study with a small sample size shows significant patient reported improvements post‐operatively, with minimal complications and re‐dislocations [90].

#### Allografts

##### Distal tibial allograft

The DTA was first introduced by Provencher et al. [69], and has emerged as an alternative to traditional coracoid or iliac crest transfers and chosen for its anatomical fit to the distal glenoid, with dense cortical bone and a native cartilaginous surface that closely conforms to the humeral head [69]. Wong and Urquhart [88] described Arthroscopic Anatomic Glenoid Reconstruction (AAGR), a subscapularis‐sparing arthroscopic technique for reconstruction of the anterior glenoid with DTA [9, 69, 87]. AAGR with DTA has since resulted in positive patient outcomes, low recurrence rates, and low complications [3, 48, 83, 87, 89]. The use of a DTA in an open or arthroscopic approach still has reported complications, albeit low (3.75%) [75], with concerns about the consistency of graft incorporation and the potential for resorption.

##### Iliac crest allograft

The implantation technique for iliac crest allografts closely resembles that of autografts, except that harvesting from the patient is not required. Few studies examine the implication of iliac crest allografts and non‐rigid fixation methods [78, 79]. This approach has highlighted good patient outcomes, with minimal rates of recurrence, and high optimal graft position and healing rates >90% [80, 92].

### Graft resorption

#### Defining and measuring graft resorption

**Figure 2:**
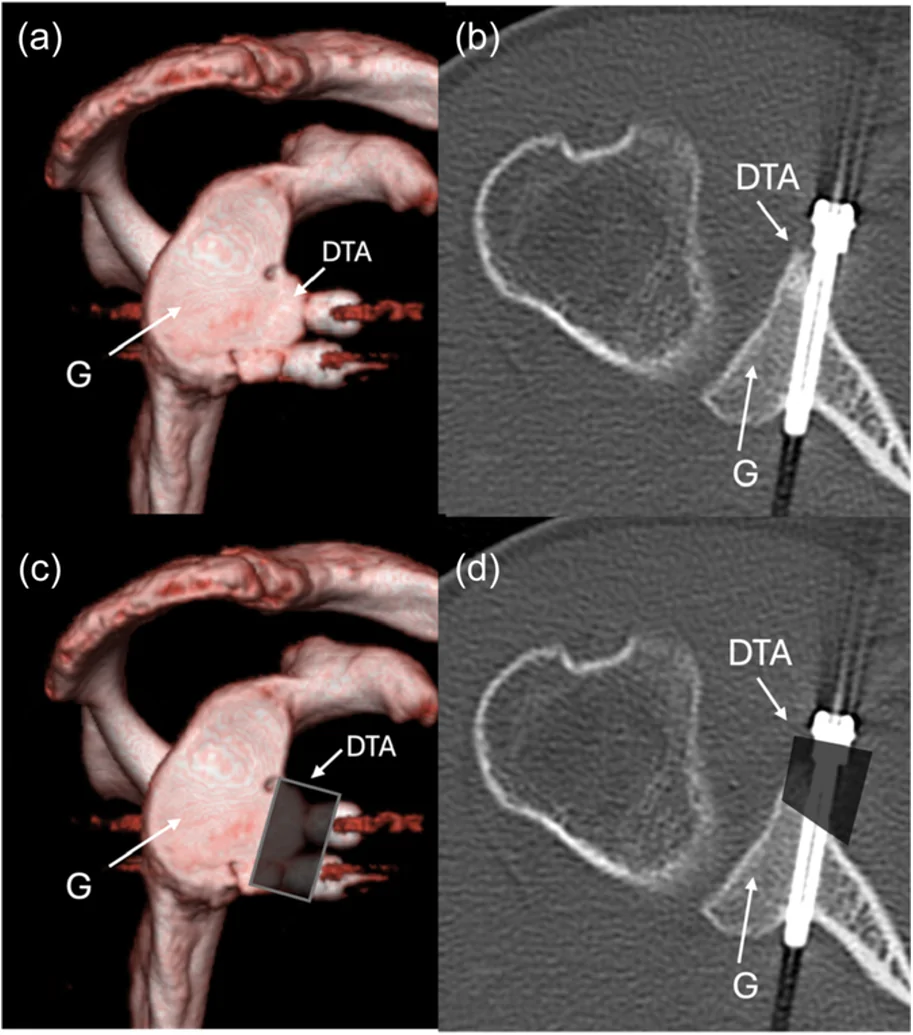
(a) Three‐dimensional computed tomography (CT) scan *en‐face* view. (b) Axial two‐dimensional CT scan view of a right shoulder demonstrating continuous cortical structure with the native glenoid. In (b) the anterior rim of the glenoid is in continuity in the axial plane, and the glenoid surface is curved and lacks discontinuity, often seen as sharp angles where the graft was inserted. (c) and (d) Estimation of where the graft would have been placed, to highlight its remodelling. DTA, distal tibia allograft; G, glenoid.

Graft resorption is a known complication of all bone grafting techniques; however, there is some confusion between normal or expected resorption (resulting in remodelling) and excessive graft resorption (resulting in graft failure). *Resorption* is the process of the breakdown and removal of bone whereas *remodelling* is the process of bone being rebuilt and reshaped [33, 34, 72]. Complete resorption is referred to as *osteolysis* [1]. Often studies report on union or non‐union of the graft as well, which outline the healing of the graft to the native glenoid. A CT scan is the gold standard measurement tool for graft resorption. Views in both the axial and *en‐face* planes are used to evaluate graft morphology, cortical continuity, and overall volume (Figure 2). Multiple techniques to meausure graft resorption exist [36, 93]. Zhu et al. [93] proposed a classification system using axial CT where resorption was assessed based on the degree of screw exposure, ranging from ‘Grade 0’ as the screw head buried in the graft to ‘Grade III’ as the screw head and shaft are completely exposed with no bone left on the glenoid neck [93].

Conversely, when suspensory fixation is used, resorption can be evaluated by comparing the dimensions of a perfect circle on immediate post‐op CT and final CT scans [14]. However, neither of these methods provide information about graft stability or indicate when graft remodelling is complete. Instead, graft stability can be inferred when the cortical outline of the glenoid surface appears continuous and anatomically restored on both the axial and *en‐face* CT scan (Figure 2) [22]. We define this radiographic appearance as indicative of full remodelling, suggesting that the graft has integrated and the glenoid structure is unlikely to undergo further change, unless a re‐dislocation occurs, impacting the remodelled anterior glenoid rim.

Research and clinical expertise point to the fact that resorption will occur to some level when using an autograft or allograft [22, 23, 59, 87]. Authors attribute resorption and remodelling following bone augmentation to Wolff′s law, a ‘hot topic’. In its simplest form, Wolff′s law states that a bone adapts to the loads under which it is placed [33, 34]. Following a bony procedure for GBL, Wolff′s law would hold true if enough load is applied at the level of the glenohumeral joint to adapt. Therefore, the surface of the glenoid will reshape to its natural form under stress [20]. In bony procedures, it is important to place the graft accurately. A medially placed graft could increase the resorption due to inadequate mechanical stress placed across the graft. Conversely, a laterally placed graft has the potential to increase the joint forces and thereby lead to arthritis. In addition to mechanical loading being a factor in graft resorption, biological factors, including poor vascularisation, immune response, and reduced osteogenic potential of the graft can influence the level of resorption [11, 38, 87].

### Autograft resorption

Graft resorption for the Latarjet with coracoid autograft transfer is variable, where complete resorption has been reported to be as low as 5% [19], and as high as 100% [23, 77, 93]. In one example, Tanaka et al. [77] reported 100% incidence of bone resorption following the Latarjet procedure with screw fixation in rugby players. By one year, all cases showed resorption at the superior screw (severe in 50%), while only 20% showed resorption at the inferior screw [77]. Moreover, open Latarjet procedures with screw fixation have reported high rates of graft union (88‐100%) [50, 76], but one study has shown about 12% non‐union [50]. When comparing these results to the arthroscopic Latarjet procedure with screw fixation, healing or union is reportedly similar [37, 50, 85], but Kordasiewicz et al. showed less non‐union (1.7%) and development of osteolysis (0%) in arthroscopic procedures [50]. However, it is important to note that while higher non‐union was reported following open Latarjet, compared to the arthroscopic Latarjet with screws, the follow‐up was more than twice as long in the open group, giving more time for resorption or osteolysis to occur.

This resorption phenomenon further differs regarding screw or suture button fixation across Latarjet procedures. Some literature supports superior graft healing and union rates when rigid‐screw fixation was used, with follow up ranging from 6 months to 3 years [15, 37, 63, 85]. However, higher resorption or osteolysis is often reported when screw fixation is used [37, 63, 85]. Di Giacomo et al. explain that increasing compression is not enough to achieve less coracoid bone graft osteolysis [35], whereas Wang et al. describe that the more flexible fixation pattern of suture buttons helps with good bone contact with less compressive stress, leading to union and, not necessarily osteolysis [85].

Following ICBGT procedures, Boehm et al. [12] in a series of 14 patients had CT imaging confirm complete graft ‘consolidation’ in all cases, with an initial overcorrection of glenoid surface area that remodelled over time toward a normal anatomic shape, indicating successful graft remodelling without significant resorption. Additionally, in a small cohort (*n* = 11), Wong et al. [64] found that six patients exhibited <25% resorption, four experienced 25%–50% resorption, and one experienced >50% resorption. These findings align with Delgado et al. [23] who reported that approximately half of their patients had >20% of graft resorption. These results suggest that while varying degrees of graft resorption are common following ICBGT, the remodelling process is often sufficient to restore glenoid anatomy. Importantly, the presence of resorption does not appear to compromise clinical outcomes, underscoring the robustness of the technique.

### Allograft resorption

Graft union and resorption are critical considerations in the success of DTA augmentation for anterior shoulder instability. In a recent meta‐analysis, Singh et al. [75] highlighted 36.47% prevalence of graft resorption across eight DTA studies. In one of the earliest clinical reports, Provencher et al. [69] described an open anatomic glenoid reconstruction with DTA in 10 patients with recurrent instability and significant GBL. At a minimum 15‐month follow‐up, all grafts appeared to be united radiographically, and no cases of significant resorption were identified, although a major limitation was that CT imaging was not systematically used. In a larger cohort of 41 patients, Provencher et al. [68] used CT imaging and revealed full graft union in 87% of patients, but 41% exhibited some degree of graft resorption, typically at the anterior‐inferior margin. Amar et al. reported low levels of resorption at short‐term (6.31 months) follow‐up of an arthroscopic DTA technique with rigid screw fixation using CT imaging, supporting DTA′s potential for stable osseointegration [3]. Specifically, full osseous union was achieved in 83% of patients, while 17% showed partial union. Importantly, resorption was minimal, with 87% of grafts maintaining >75% of original volume. This study highlighted the role of arthroscopic technique and rigid fixation in promoting graft stability and minimising resorptive change [3]. Similarly, Wong et al. [89] reported on 35 patients with CT assessment at a mean of 12.8 months. They observed complete graft union in 91% of patients and partial resorption in 14%, with only 6% demonstrating >25% volume loss [89].

In comparison, resorption following fixation of iliac crest allograft, either using screws or non‐rigid suture button fixation, reveal high resorption rates [11, 23, 29, 92]. In Espejo Reina et al. and Delgado et al., with study samples of 14 and 10, respectively, arthroscopic iliac crest allograft fixation using suture buttons resulted in over 90% of the population having >20% of resorption and high rates of osteolysis [23, 29]. When screws are used for fixation of the iliac crest allograft, Boehm et al. reported ‘excessive’ graft resorption [11], and Mascarenhas et al. highlighted that 80% had osseous union at six months follow up and only 20% demonstrating some degree of osteolysis [57]. The lower resorption rates observed with DTAs compared to iliac crest allografts may be attributed to the osteochondral composition of the DTA, which allows restoration of the articular hyaline cartilage surface, in contrast to the osseous iliac crest graft [17, 73].

Recurrent instability remains low when using DTA in an open or arthroscopic approach [68, 75, 83, 89]. Tucker et al. reported a significantly lower rate of recurrent instability in DTA patients (5%) compared to those undergoing Bankart repair (27%), suggesting that even in the presence of partial resorption, clinical stability can be maintained [83]. In the short to medium term (<2 years) Wong et al. and Singh et al. found that functional scores were high, and recurrence rates remained low, again when partial resorption was observed [75, 89]. Boehm et al. concur with these results showing no re‐dislocations [11]. This finding may be due to the restoration of the glenoid arc and capsulolabral tension, which remain protective against dislocation [11]. Furthermore, it has been suggested that re‐dislocation rates remain low despite the high resorption, particularly seen in the iliac crest allograft due to the formation of stabilising scar tissue [11]. However, severe or progressive resorption may compromise the graft′s ability to prevent humeral head translation, particularly in high‐demand athletes [32]. In these DTA and iliac crest allograft studies, clinical outcomes remained favourable, with minimal subluxations or dislocations throughout the studies leading authors to conclude that resorption did not correlate with clinical failure or recurrence [11, 29, 68, 87].

### Allograft vs. autograft

Comparisons between graft types have also shed light on their behaviour over time. Wong et al. [87] performed a radiographic analysis comparing coracoid autograft and DTA reconstructions. In the DTA group, mean graft volume decreased by 11.8% from immediate postoperative imaging to final follow‐up (average 13.4 months), while the coracoid group showed only a 3.2% decrease. In a matched cohort study, Frank et al. [30] found radiographic evidence of partial resorption in 39% of DTA grafts versus only 11% of Latarjet grafts. Similarly, in a direct comparison study, Delgado et al. showed allograft bone (iliac crest) had greater resorption than that of the iliac crest autograft or open and arthroscopic Latarjet procedures [23]. These findings underscore the importance of understanding the biological behaviour of allograft and autograft bone, particularly the remodelling potential of non‐vascularised constructs such as distal tibial or iliac crest grafts. While both allograft‐based (e.g., DTA) and autograft‐based (e.g., Latarjet) procedures are susceptible to osteolysis [36, 49], the osteochondral nature of DTA may better replicate the native glenoid contour and reduce joint contact pressures [31]. In contrast, the coracoid graft benefits from partial preservation of vascularity through the conjoint tendon, which may mitigate resorption at its inferior aspect. Nonetheless, superior regions of the coracoid may remain prone to resorption due to limited vascularisation and mechanical loading. Ultimately, both techniques share comparable remodelling dynamics and remain vulnerable to partial graft resorption despite differing biological and mechanical characteristics.

### Distal tibia allograft – remodelling timeline and factors

Biomechanical and imaging‐based studies offer further context on the timeline of the remodelling nature of bone grafts in the treatment of anterior shoulder instability. The application of Wolff′s law in DTA reconstruction was recently further explored by Dawe et al. [22], who demonstrated that resorption following AAGR with DTA results in a return to anatomic glenoid width by an average 6.9 months post‐operation. Their findings highlight that graft remodelling, consistent with Wolff′s law, plays a key role in *restoring* the native glenoid size and architecture.

Ultimately, radiographic studies have demonstrated that allografts undergo a natural remodelling process upon implantation, including vascular invasion, osteoclastic activity, and eventual incorporation [34]. However, this process is not always complete. Partial or even significant resorption of DTA has been documented, particularly in patients with poor graft fixation or suboptimal mechanical loading due to a medialized graft [75, 89]. Due to this variability, we highlight the importance of reporting final anterior‐posterior width of the graft and glenoid on a post‐operative CT at a minimum of 7 months following surgery [22], to define *remodelling*.

#### Understanding remodelling

While authors have reported rates of resorption and estimated amounts of bone loss following that resorption, less is reported on remodelling. We note from Wolff′s law that the bone will remodel based on the stress that is applied to it, and therefore, we would expect the glenoid to remodel back to the estimated native size, as determined using the superior‐inferior and anterior‐posterior methodology [70]. We define *remodelling* as *stabilised or finished when the axial and en‐face views have complete normal cortical structure* (Figure 2a,b) [22]. From these figures of a remodelled DTA, the bone graft is completely healed and remodelled back to both the size and shape of the normal glenoid. We suggest that reporting anterior‐posterior glenoid and graft dimensions are more clinically meaningful than focusing solely on the degree of resorption, as often described previously. The extent of resorption is largely influenced by the initial graft size, and oversized grafts tend to undergo more resorption but ultimately remodel to produce a glenoid of normal dimensions. We aim to discuss if and how we can influence remodelling and what we have learned through clinical and research applications of arthroscopic bone grafting with DTA for shoulder instability with anterior GBL.

### Remodelling timeline and factors

**Figure 3:**
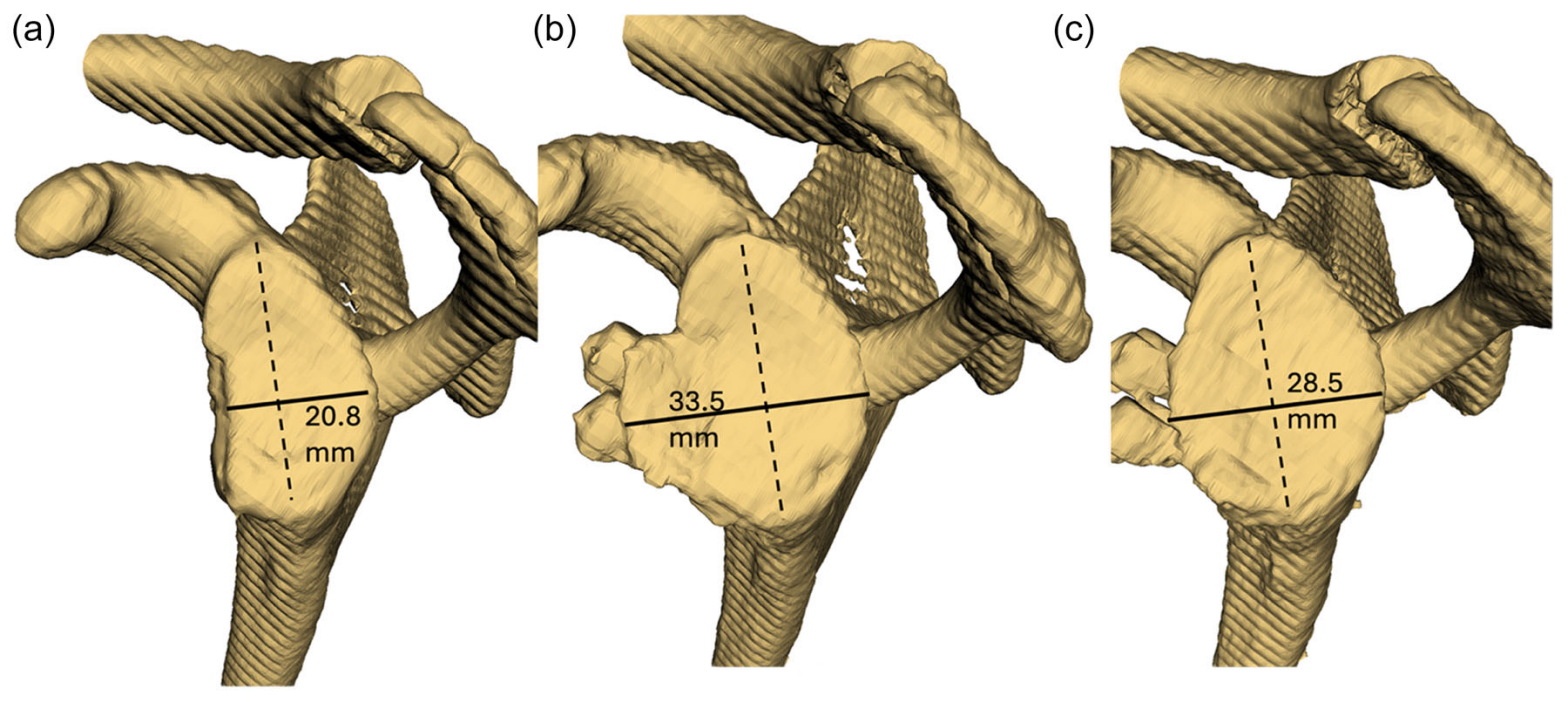
(a) Pre‐operative, (b) early (4 months) post‐operative computed tomography (CT) scan, and (c) 13‐month post‐operative CT scan demonstrating increased remodelling over time and increased hardware prominence at final graft remodelling with dotted lines representing superior‐inferior measurement as 37.3 mm. Solid lines represent anterior‐posterior glenoid width measurements.

The use of DTA in the management of anterior shoulder instability has evolved significantly since its initial open adaptation by Provencher et al. [69]. Through the development of AAGR with DTA [88], the early stages of DTA implementation involved larger grafts being used under the rationale that greater bone loss warranted more substantial bony reconstruction to restore glenoid concavity and stability (see Figure 3a). Early postoperative CT scans of these patients, typically obtained around 3‐months post‐operatively, did not show clear evidence of graft integration or remodelling (Figure 3b). This period, characterised by relatively static imaging findings, coincided with minimal patient symptoms highlighting that patients′ shoulders were now considered stable following this procedure. It wasn′t until closer to the 12‐month follow‐up that some patients began reporting symptoms potentially related to hardware irritation, prompting further imaging (Figure 3c).

**Figure 4:**
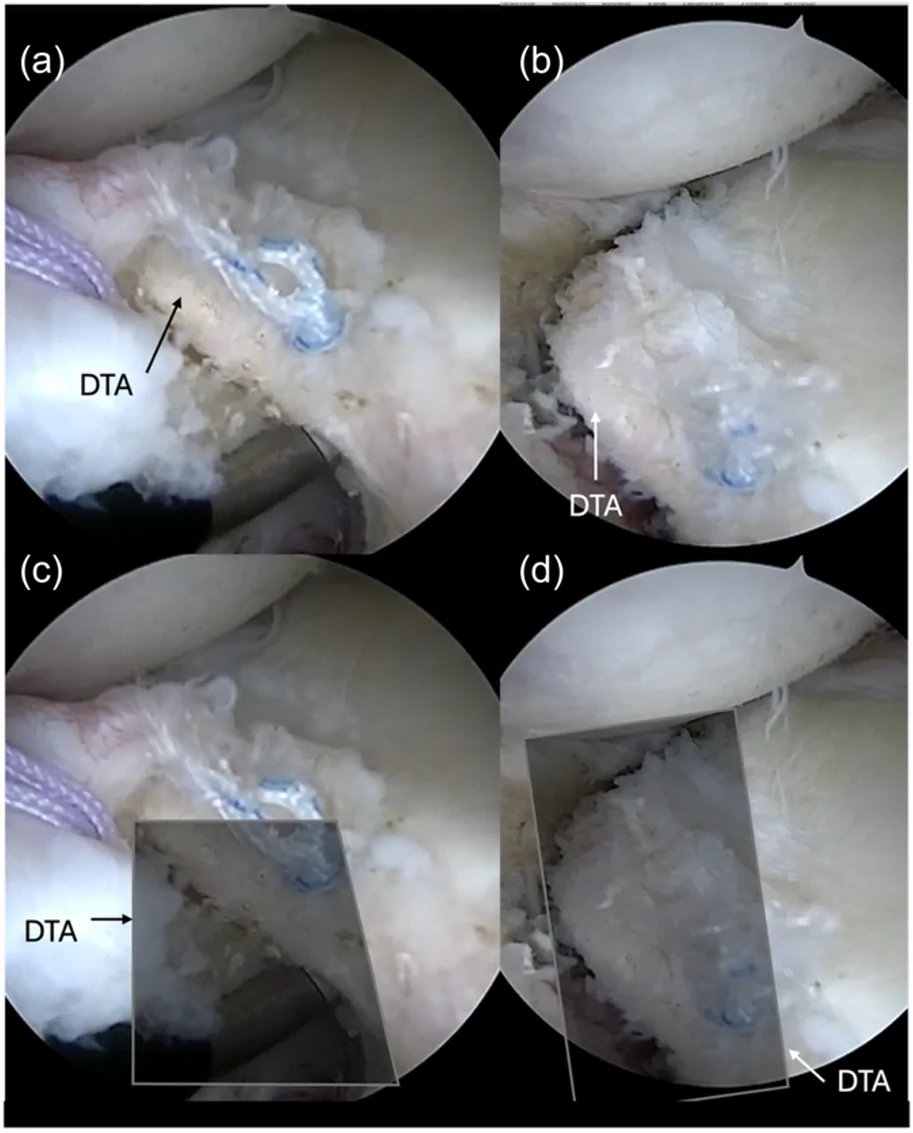
Remodelled distal tibia allograft (DTA) demonstrating (a) triangular remodelled shape axially and (b) continuity of the cortex and healing of the Bankart repair over the graft. (c) and (d) Overlay estimated graft placement to highlight remodelling.

Follow‐up CT scans of these initial patients at the 12‐month mark revealed more substantial remodelling of the graft (see Figure 3c), with progressive rounding of the graft margins and cortical continuity suggestive of osseous integration. Axial images revealed restoration of the native glenoid concavity, with the grafts adopting a triangular morphology, which further indicated the remodelling process. In some cases, the initially oversized DTA grafts led to prominent screw heads becoming exposed as the bone remodelled (Figure 3c), contributing to anterior shoulder pain and stiffness. This occasionally necessitated secondary arthroscopic procedures, including hardware removal. In patients who required arthroscopic screw removal, a well‐integrated triangular shaped graft remained under the Bankart repair, confirming successful osseous integration (Figure 4). These findings prompted a shift toward using smaller, more anatomically matched grafts, particularly as clinical outcomes remained favourable despite partial graft resorption and re‐dislocation rates remained low [3, 83, 89]. This experience raised important biomechanical and biological considerations, including: how much graft is truly necessary to restore function, and to what extent can resorption be tolerable without compromising long‐term joint stability?

**Figure 5:**
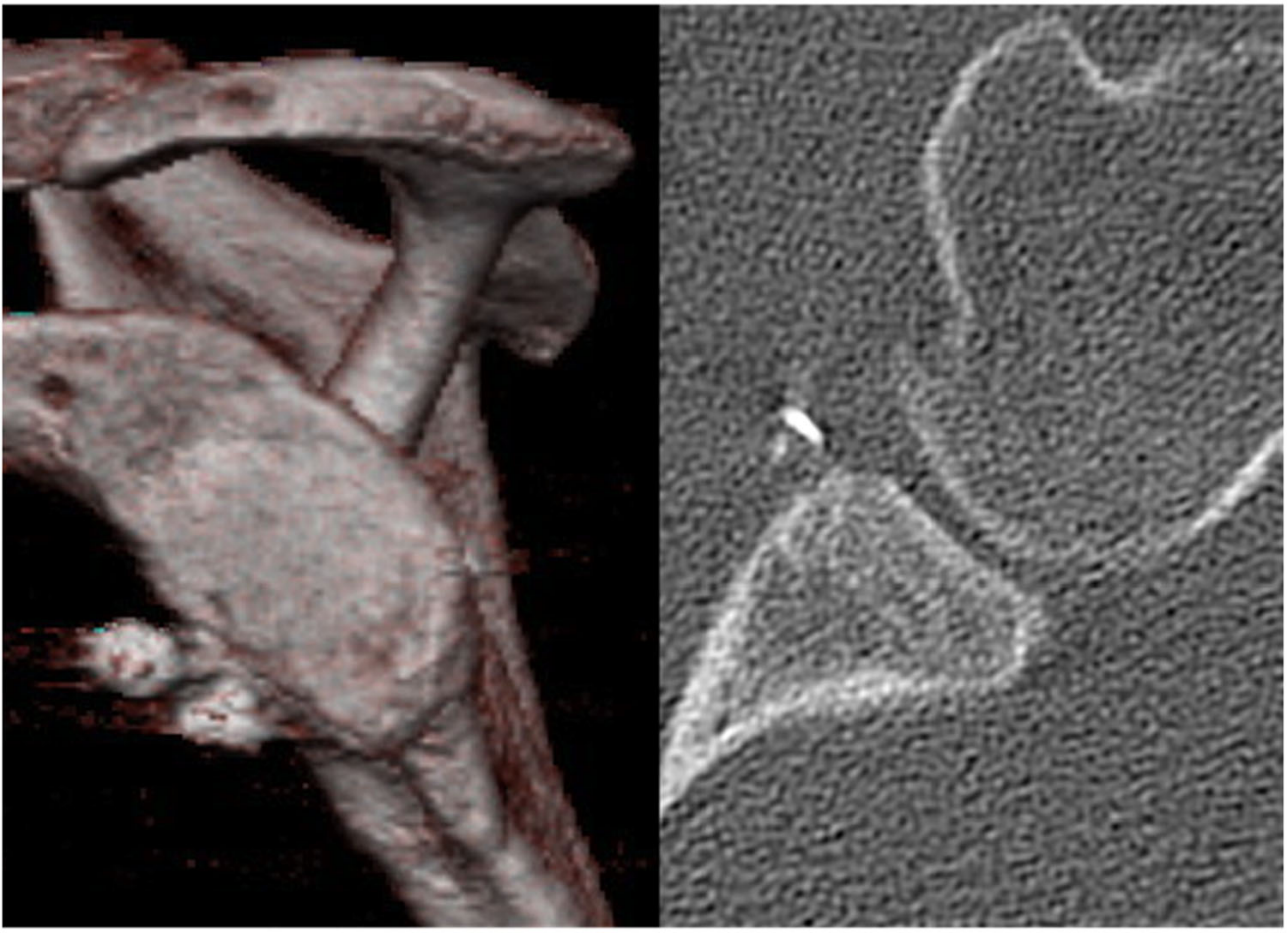
Suspensory fixation method demonstrating high level of resorption where remodelled glenoid anterior‐posterior width remains smaller than estimated glenoid width.

Suspensory fixation has gained traction, largely for its ease of use in teaching environments and potential to minimise hardware‐related complications. However, comparing DTA fixation with screws and suspensory methods showed that on average, suspensory fixation results in greater bone resorption and subsequently smaller postoperative glenoid anterior‐posterior width than screw fixation (Figure 5) [65]. Additionally, these findings corresponded with an increased re‐dislocation rate compared to screw fixation. This finding contrasts with good long‐term outcomes achieved with suspensory fixation in the Latarjet procedure [24]. We theorise that due to the slower osseointegration of allograft compared to autograft, graft resorption and remodelling may occur before full incorporation to the anterior glenoid neck. This early remodelling could lead to de‐tensioning of the suspensory fixation, resulting in medial graft displacement and severe resorption. Moreover, suspensory fixation does not handle shear forces well, making it very difficult to recreate the glenoid′s concavity that normally helps load the graft and drive remodelling through Wolff′s law. Over time, this fixation method likely leads to graft erosion, increased subluxation events, recurrent bone loss, and possible redislocation. Despite these findings, questions persist regarding the long‐term implications of graft resorption and hardware design. For instance, does the type of screw or even its positioning impact remodelling? Could surface‐treated or bioresorbable screws mitigate complications without sacrificing stability?

Looking ahead, a key question remains: can we actively influence graft remodelling through surgical technique? With DTA, surgeons have control over both fixation method and graft size, variables that may shape the biological response and long‐term outcomes. In cases where full graft resorption occurs without recurrent instability, it is often because there was no true bone loss to begin with. This nuance underscores the importance of appropriate patient selection and highlights the potential for tailoring surgical technique, such as opting for specific fixation methods or customising the graft size to optimise remodelling while minimising complications. As the field moves forward, refining these decisions could enhance surgical outcomes and personalise care for patients with shoulder instability.

## CONCLUSION

Bony augmentation procedures may be considered in the setting of anterior shoulder instability with critical GBL. While the Latarjet procedure remains a widely used autograft technique, complications and graft resorption remain concerns. Meanwhile, DTA represents a biomechanically favourable alternative with excellent clinical outcomes. To better characterise graft remodelling, final glenoid width should be assessed with CT scans at least seven months post‐operatively to show when the cortex has remodelled to the native glenoid. Future studies should report not only resorption but also remodelling patterns across techniques and graft types and determine how these relate to long‐term outcomes and clinical failure.

## AUTHOR CONTRIBUTIONS

All authors contributed to the conceptualisation and development of the manuscript. Sarah Remedios conducted the literature review and synthesised the evidence. The first draft was written by Sarah Remedios, and all authors critically reviewed and revised subsequent versions. All authors approved the final manuscript.

## CONFLICT OF INTEREST STATEMENT

Sarah Remedios declare that they have no known competing financial interests or personal relationships that could have appeared to influence the work reported in this paper. Jillian Karpyshyn declare that they have no known competing financial interests or personal relationships that could have appeared to influence the work reported in this paper. Dr. Ivan Wong reports a relationship with DePuy Mitek Inc that includes: consulting or advisory and speaking and lecture fees. Dr. Ivan Wong reports a relationship with Smith and Nephew Inc that includes: consulting or advisory and speaking and lecture fees. Dr. Ivan Wong reports a relationship with CONMED Corp that includes: consulting or advisory and speaking and lecture fees. Dr. Ivan Wong reports a relationship with Bioventus LLC that includes: speaking and lecture fees. Dr. Ivan Wong is an editorial board member for the American Journal of Sports Medicine (AJSM) and Arthroscopy: The Journal of Arthroscopic and Related Surgery (ARTH), and The HIVE Musculoskeletal Journal; and is a board of committee member for AANA, ISAKOS and AAC. Is an associate editor for the Orthopaedic Journal of Sports Medicine (OJSM). If there are other authors, they declare that they have no known competing financial interests or personal relationships that could have appeared to influence the work reported in this paper.

## ETHICS STATEMENT

There is no institutional review board (IRB) approval required for this study.

## References

[1] Agarwal S . Osteolysis—basic science, incidence and diagnosis. Curr Orthop. 2004;18:220–231.

[2] Alkaduhimi H , Willigenburg NW , Wessel RN , Wolterbeek N , Veen EJD , Koorevaar RCT , et al. Ninety‐day complication rate based on 532 Latarjet procedures in Dutch hospitals with different operation volumes. J Shoulder Elbow Surg. 2023;32:1207–1213. 10.1016/j.jse.2022.11.015 36586507

[3] Amar E , Konstantinidis G , Coady C , Wong IH . Arthroscopic treatment of shoulder instability with glenoid bone loss using distal tibial allograft augmentation: safety profile and short‐term radiological outcomes. Orthop J Sports Med. 2018;6:232596711877450. 10.1177/2325967118774507 PMC5971393 29854863

[4] An VVG , Sivakumar BS , Phan K , Trantalis J . A systematic review and meta‐analysis of clinical and patient‐reported outcomes following two procedures for recurrent traumatic anterior instability of the shoulder: Latarjet procedure vs. Bankart repair. J Shoulder Elbow Surg. 2016;25:853–863. 10.1016/j.jse.2015.11.001 26809355

[5] Auffarth A , Schauer J , Matis N , Kofier B , Hitzl W , Resch H . The J‐bone graft for anatomical glenoid reconstruction in recurrent posttraumatic anterior shoulder dislocation. Am J Sports Med. 2008;36:638–647. 10.1177/0363546507309672 18006673

[6] Aurich M , Hofmann GO , Best N . Clinical outcome and return to sports activity after surgical treatment for recurrent shoulder instability with a modified Latarjet procedure. Orthop Traumatol Surg Res. 2021;107:102977. 10.1016/j.otsr.2021.102977 34091084

[7] Bessière C , Trojani C , Carles M , Mehta SS , Boileau P . The open latarjet procedure is more reliable in terms of shoulder stability than arthroscopic bankart repair. Clin Orthop Relat Res. 2014;472:2345–2351. 10.1007/s11999-014-3550-9 PMC4079884 24615422

[8] Bhatia S , Saigal A , Frank RM , Bach BR , Cole BJ , Romeo AA , et al. Glenoid diameter is an inaccurate method for percent glenoid bone loss quantification: analysis and techniques for improved accuracy. Arthrosc ‐ J Arthrosc Relat Surg. 2015;31:608–614.e1. 10.1016/j.arthro.2015.02.020 25842231

[9] Bhatia S , Van Thiel GS , Gupta D , Ghodadra N , Cole BJ , Bach BR , et al. Comparison of glenohumeral contact pressures and contact areas after glenoid reconstruction With Latarjet or Distal Tibial Osteochondral Allografts. Am J Sports Med. 2013;41:1900–1908. 10.1177/0363546513490646 23775244

[10] Bockmann B , Nebelung W , Gröger F , Leuzinger J , Agneskirchner J , Brunner U , et al. The arthroscopic treatment of anterior shoulder instability with glenoid bone loss shows similar clinical results after Latarjet procedure and iliac crest autograft transfer. Knee Surg Sports Traumatol Arthrosc. 2023;31:4566–4574. 10.1007/s00167-023-07480-2 37386197

[11] Boehm E , Minkus M , Moroder P , Scheibel M . Massive graft resorption after iliac crest allograft reconstruction for glenoid bone loss in recurrent anterior shoulder instability. Arch Orthop Trauma Surg. 2020;140:895–903. 10.1007/s00402-020-03380-z 32090290

[12] Boehm E , Minkus M , Moroder P , Scheibel M . Arthroscopic iliac crest bone grafting in recurrent anterior shoulder instability: minimum 5‐year clinical and radiologic follow‐up. Knee Surg Sports Traumatol Arthrosc. 2021;29:266–274. 10.1007/s00167-020-05986-7 PMC7862210 32285158

[13] Boileau P , Saliken D , Gendre P , Seeto BL , d'Ollonne T , Gonzalez J‐F , et al. Arthroscopic latarjet: suture‐button fixation is a safe and reliable alternative to screw fixation. Arthrosc ‐ J Arthrosc Relat Surg. 2019;35:1050–1061. 10.1016/j.arthro.2018.11.012 30857907

[14] Boissinot T , Baltassat A , Barret H , Girard M , Mansat P , Bonnevialle N . Arthroscopic Bankart repair augmented with glenoid bone dry allograft. JSES Int. 2025;9:40–45. 10.1016/j.jseint.2024.09.008 PMC11784495 39898207

[15] Bonnevialle N , Girard M , Dalmas Y , Martinel V , Faruch M , Mansat P . Short‐term bone fusion with arthroscopic double‐button latarjet versus open‐screw latarjet. Am J Sports Med. 2021;49:1596–1603. 10.1177/03635465211001095 33830790

[16] Briggs DV , Hurley ET , Warren E , Amanah AY , Levin JM , Lau BC , et al. Bone block options for treating glenoid bone loss and glenohumeral instability: a systematic review. Shoulder Elbow. 2025;17:543–553. 10.1177/17585732241293763 PMC11559950 39545005

[17] Bugbee WD , Richard Convery F . Osteochondral allograft transplantation. Clin Sports Med. 1999;18:67–75. 10.1016/s0278-5919(05)70130-7 10028117

[18] Cerciello S , Corona K , Morris BJ , Santagada DA , Maccauro G . Early outcomes and perioperative complications of the arthroscopic latarjet procedure: systematic review and meta‐analysis. Am J Sports Med. 2019;47:2232–2241. 10.1177/0363546518783743 30067066

[19] Chillemi C , Guerrisi M , Paglialunga C , Salate Santone F , Osimani M . Latarjet procedure for anterior shoulder instability: a 24‐year follow‐up study. Arch Orthop Trauma Surg. 2021;141:189–196. 10.1007/s00402-020-03426-2 32221703

[20] Cole BJ , Millett PJ , Romeo AA , Burkhart SS , Andrews JR , Dugas JR , et al. Arthroscopic treatment of anterior glenohumeral instability: indications and techniques. Instr Course Lect. 2004;53:545–558. 15116643

[21] Colegate‐Stone TJ , van der Watt C , de Beer JF . Evaluation of functional outcomes and complications following modified Latarjet reconstruction in athletes with anterior shoulder instability. Shoulder Elbow. 2015;7:168–173. 10.1177/1758573215578588 PMC4935152 27582973

[22] Dawe N , Ma J , Wong I . Allograft resorption following arthroscopic anatomic glenoid reconstruction is part of remodeling to restore the native glenoid size and shape 6.9 months postoperatively. Arthrosc J Arthrosc Relat Surg. 2025;41:3846–3853. 10.1016/j.arthro.2025.03.048 40158823

[23] Delgado C , Calvo E , Díaz Heredia J , Cañete P , García Navlet M , Ruiz Ibán MA . Graft position, healing, and resorption in anterior glenohumeral instability: a comparison of 4 glenoid augmentation techniques. Orthop J Sports Med. 2024;12:23259671241253163. 10.1177/23259671241253163 PMC11151773 38840788

[24] Descamps J , Greco V , Chelli M , Boileau P . The arthroscopically guided bristow‐latarjet procedure with cortical button fixation: a minimum 10‐year follow‐up. Am J Sports Med. 2024;52:2815–2825. 10.1177/03635465241263590 39221758

[25] Dickens JF , Owens BD , Cameron KL , DeBerardino TM , Masini BD , Peck KY , et al. The effect of subcritical bone loss and exposure on recurrent instability after arthroscopic bankart repair in intercollegiate american football. Am J Sports Med. 2017;45:1769–1775. 10.1177/0363546517704184 28474965

[26] Dickens JF , Slaven SE , Cameron KL , Pickett AM , Posner M , Campbell SE , et al. Prospective evaluation of glenoid bone loss after first‐time and recurrent anterior glenohumeral instability events. Am J Sports Med. 2019;47:1082–1089. 10.1177/0363546519831286 30943084

[27] Dumont GD , Fogerty S , Rosso C , Lafosse L . The arthroscopic latarjet procedure for anterior shoulder instability: 5‐year minimum follow‐up. Am J Sports Med. 2014;42:2560–2566. 10.1177/0363546514544682 25117728

[28] Eden R . Zur Operation der habituellen Schulterluxation unter Mitteilung eines neuen verfahrens bei Abriß am inneren Pfannenrande. Dtsch Z Chir. 1918;144:269–280.

[29] Espejo Reina MJ , Delgado C , Ruiz Díaz R , Díaz Heredia J , Asenjo Gismero C , Ruiz Ibán MA . Outcomes of an anterior bone block technique with iliac crest allograft for the management of anteroinferior shoulder instability with subcritical glenoid defects. J Shoulder Elbow Surg. 2025;34:e1060–e1069. 10.1016/j.jse.2025.02.022 40118439

[30] Frank RM , Romeo AA , Richardson C , Sumner S , Verma NN , Cole BJ , et al. Outcomes of latarjet versus distal tibia allograft for anterior shoulder instability repair: a matched cohort analysis. Am J Sports Med. 2018;46:1030–1038. 10.1177/0363546517744203 29389219

[31] Frank RM , Shin J , Saccomanno MF , Bhatia S , Shewman E , Bach Jr. BR , et al. (2014). Comparison of glenohumeral contact pressures and contact areas after posterior glenoid reconstruction with an iliac crest bone graft or distal tibial osteochondral allograft. Am J Sports Med. 42:2574–2582. 10.1177/0363546514545860 25193887

[32] Friedman LGM , Lafosse L , Garrigues GE . Global perspectives on management of shoulder instability. Orthop Clin North Am. 2020;51:241–258. 10.1016/j.ocl.2019.11.008 32138862

[33] Frost HM . Wolff′s Law and bone′s structural adaptations to mechanical usage: an overview for clinicians. Angle Orthod. 1994;64:175–188. 10.1043/0003-3219(1994)064<0175:WLABSA>2.0.CO;2 8060014

[34] Frost HM . A 2003 update of bone physiology and Wolff′s law for clinicians. Angle Orthod. 2004;74:3–15. 10.1043/0003-3219(2004)074<0003:AUOBPA>2.0.CO;2 15038485

[35] Giacomo G , de Gasperis N , De Vita A , Francone M , Lin BH , Mastantuono M , et al. Coracoid bone graft osteolysis after Latarjet procedure: a comparison study between two screws standard technique vs mini‐plate fixation. Int J Shoulder Surg. 2013;7:1–6. 10.4103/0973-6042.109877 PMC3707330 23858288

[36] Di Giacomo G , de Gasperis N , Costantini A , De Vita A , Beccaglia MAR , Pouliart N . Does the presence of glenoid bone loss influence coracoid bone graft osteolysis after the Latarjet procedure? A computed tomography scan study in 2 groups of patients with and without glenoid bone loss. J Shoulder Elbow Surg. 2014;23:514–518. 10.1016/j.jse.2013.10.005 24406124

[37] Girard M , Dalmas Y , Martinel V , Mansat P , Bonnevialle N . Arthroscopic latarjet with cortical buttons versus open latarjet with screws: a short‐term comparative study. Am J Sports Med. 2022;50:3326–3332. 10.1177/03635465221120076 36053060

[38] Goldberg VM , Stevenson S . Natural history of autografts and allografts. Clin Orthop Relat Res. 1987;225:7–16. 3315383

[39] Griesser MJ , Harris JD , McCoy BW , Hussain WM , Jones MH , Bishop JY , et al. Complications and re‐operations after Bristow‐Latarjet shoulder stabilization: a systematic review. J Shoulder Elbow Surg. 2013;22:286–292. 10.1016/j.jse.2012.09.009 23352473

[40] Gupta A , Delaney R , Petkin K , Lafosse L . Complications of the Latarjet procedure. Curr Rev Musculoskelet Med. 2015;8:59–66. 10.1007/s12178-015-9258-y PMC4596182 25644052

[41] Hamamoto JT , Leroux T , Chahla J , Bhatia S , Higgins JD , Romeo AA , et al. Assessment and evaluation of glenoid bone loss. Arthrosc Tech. 2016;5:e947–e951. 10.1016/j.eats.2016.04.027 PMC5040627 27709063

[42] Huddleston H , Credille K , Wang Z , Cregar W , Lansdown DA , Chahla J , et al. Current methods used to evaluate glenoid bone loss: a survey of orthopaedic surgeons. Orthop J Sports Med. 2025;13:23259671241288163. 10.1177/23259671241288163 PMC11833853 39968413

[43] Huijsmans PE , Haen PS , Kidd M , Dhert WJ , van der Hulst VPM , Willems WJ . Quantification of a glenoid defect with three‐dimensional computed tomography and magnetic resonance imaging: a cadaveric study. J Shoulder Elbow Surg. 2007;16:803–809. 10.1016/j.jse.2007.02.115 18061117

[44] Hurley ET , Ben Ari E , Lorentz NA , Mojica ES , Colasanti CA , Matache BA , et al. Both open and arthroscopic latarjet result in excellent outcomes and low recurrence rates for anterior shoulder instability. Arthrosc Sports Med Rehabil. 2021;3:e1955–e1960. 10.1016/j.asmr.2021.09.017 PMC8689257 34977653

[45] Hybbinette S . On the transplantation of a bone fragment to remedy recurrent dislocations of the shoulder: observations and operative results. Acta Chir Scand. 1932;71:26.

[46] Itoi E , Lee S‐B , Berglund LJ , Berge LL , An K‐N . The effect of a glenoid defect on anteroinferior stability of the shoulder after bankart repair: a cadaveric study. J Bone Joint Surg Am. 2000;82:35–46. 10.2106/00004623-200001000-00005 10653082

[47] Jackson GR , Brusalis CM , Schundler SF , Sachdev D , Obioha OA , McCormick JR , et al. Isolated primary latarjet procedures for anterior shoulder instability results in high rates of graft resorption and glenohumeral degenerative changes with low rates of failure at a minimum 2‐year follow‐up: a systematic review. Arthrosc ‐ J Arthrosc Relat Surg. 2024;40:581–591.e1. 10.1016/j.arthro.2023.05.024 37270111

[48] Karpyshyn J , Murphy R , Sparavalo S , Ma J , Wong I . Clinical and radiographic outcomes of primary versus revision arthroscopic anatomic glenoid reconstruction with distal tibial allograft for anterior shoulder instability with bone loss. J Shoulder Elbow Surg. 2024;33:2867–2877. 10.1016/j.jse.2024.04.005 38825225

[49] Kee YM , Kim JY , Kim HJ , Sinha S , Rhee Y‐G . Fate of coracoid grafts after the Latarjet procedure: will be analogous to the original glenoid by remodelling. Knee Surg Sports Traumatol Arthrosc. 2018;26:926–932. 10.1007/s00167-017-4808-z 29198018

[50] Kordasiewicz B , Kicinski M , Małachowski K , Wieczorek J , Chaberek S , Pomianowski S . Comparative study of open and arthroscopic coracoid transfer for shoulder anterior instability (Latarjet)—computed tomography evaluation at a short term follow‐up. Part II. Int Orthop. 2018;42:1119–1128. 10.1007/s00264-017-3739-0 29299654

[51] Kraus N , Amphansap T , Gerhardt C , Scheibel M . Arthroscopic anatomic glenoid reconstruction using an autologous iliac crest bone grafting technique. J Shoulder Elbow Surg. 2014;23:1700–1708. 10.1016/j.jse.2014.03.004 24930839

[52] Lacheta L , Herbst E , Voss A , Braun S , Jungmann P , Millett PJ , et al. Insufficient consensus regarding circle size and bone loss width using the ratio—‘best fit circle’—method even with three‐dimensional computed tomography. Knee Surg Sports Traumatol Arthrosc. 2019;27:3222–3229. 10.1007/s00167-019-05391-9 30725122

[53] Lansdown DA , Wang K , Yanke AB , Nicholson GP , Cole BJ , Verma NN . A flat anterior glenoid corresponds to subcritical glenoid bone loss. Arthrosc ‐ J Arthrosc Relat Surg. 2019;35:1788–1793. 10.1016/j.arthro.2018.12.034 31060758

[54] Latarjet M . Treatment of recurrent dislocation of the shoulder. Lyon Chir. 1954;49:994–997. 13234709

[55] Makovicka J , Rodriguez M , Hassebrock J , Chung A , Tokish J . Validation of the ‘best‐fit circle’ for bone loss based upon glenoid height. Arthrosc ‐ J Arthrosc Relat Surg. 2021;37:e83–e84.

[56] Malahias M‐A , Chytas D , Raoulis V , Chronopoulos E , Brilakis E , Antonogiannakis E . Iliac crest bone grafting for the management of anterior shoulder instability in patients with glenoid bone loss: a systematic review of contemporary literature. Sports Med Open. 2020;6:12. 10.1186/s40798-020-0240-x PMC7013021 32048101

[57] Mascarenhas R , Raleigh E , McRae S , Leiter J , Saltzman B . Iliac crest allograft glenoid reconstruction for recurrent anterior shoulder instability in athletes: surgical technique and results. Int J Shoulder Surg. 2014;8:127–132. 10.4103/0973-6042.145269 PMC4262868 25538432

[58] Metais P , Clavert P , Barth J , Boileau P , Broszka R , Nourissat G , et al. Preliminary clinical outcomes of Latarjet‐Patte coracoid transfer by arthroscopy vs. open surgery: prospective multicentre study of 390 cases. Orthop Traumatol Surg Res. 2016;102:S271–S276. 10.1016/j.otsr.2016.08.003 27771428

[59] Moroder P , Blocher M , Auffarth A , Hoffelner T , Hitzl W , Tauber M , et al. Clinical and computed tomography–radiologic outcome after bony glenoid augmentation in recurrent anterior shoulder instability without significant glenoid bone loss. J Shoulder Elbow Surg. 2014;23:420–426. 10.1016/j.jse.2013.07.048 24075998

[60] Moroder P , Damm P , Wierer G , Böhm E , Minkus M , Plachel F , et al. Challenging the current concept of critical glenoid bone loss in shoulder instability: does the size measurement really tell it all? Am J Sports Med. 2019;47:688–694. 10.1177/0363546518819102 30640513

[61] Moroder P , Kathi T , Lacheta L , Karpinski K , Paksoy A , Akgün D . Arthroscopic bone block cerclage technique using a tricortical scapular spine autograft for glenoid reconstruction in patients with anterior shoulder instability. Arthrosc Tech. 2022;11:e379–e383. 10.1016/j.eats.2021.11.004 PMC8897580 35256979

[62] Moroder P , Schulz E , Wierer G , Auffarth A , Habermeyer P , Resch H , et al. Neer Award 2019: Latarjet procedure vs. iliac crest bone graft transfer for treatment of anterior shoulder instability with glenoid bone loss: a prospective randomized trial | Elsevier Enhanced Reader. J Shoulder Elbow Surg. 2019;28:1298–1307. 10.1016/j.jse.2019.03.035 31129017

[63] Neyton L , Barth J , Nourissat G , Métais P , Boileau P , Walch G , et al. Arthroscopic latarjet techniques: graft and fixation positioning assessed with 2‐dimensional computed tomography is not equivalent with standard open technique. Arthrosc ‐ J Arthrosc Relat Surg. 2018;34:2032–2040. 10.1016/j.arthro.2018.01.054 29789246

[64] Oldfield M , Burns J , Wong I . Arthroscopic glenoid bone augmentation using iliac crest autograft is safe and effective for anterior shoulder instability with bone loss. Arthrosc Sports Med Rehabil. 2021;3:e1671–e1677. 10.1016/j.asmr.2021.07.023 PMC8689205 34977619

[65] Pancura D , Licht F , Wong I . Screw fixation has better outcomes, lower incidence of redislocation, and lower bone resorption than button fixation for arthroscopic anatomic glenoid reconstruction with distal tibia allograft: a matched cohort analysis. Arthrosc ‐ J Arthrosc Relat Surg. 2025(25)00155–0. 10.1016/j.arthro.2025.02.034 40056943

[66] Piasecki DP , Verma NN , Romeo AA , Levine WN , Bach BR , Provencher MT . Glenoid bone deficiency in recurrent anterior shoulder instability: diagnosis and management. J Am Acad Orthop Surg. 2009;17:482–493. 10.5435/00124635-200908000-00002 19652030

[67] Provencher CMT , Bhatia S , Ghodadra NS , Grumet RC , Bach BR , Dewing LCB , et al. Recurrent shoulder instability: current concepts for evaluation and management of glenoid bone loss. J Bone Jt Surg. 2010;92:133–151. 10.2106/JBJS.J.00906 21123597

[68] Provencher MT , Frank RM , Golijanin P , Gross D , Cole BJ , Verma NN , et al. Distal tibia allograft glenoid reconstruction in recurrent anterior shoulder instability: clinical and radiographic outcomes. Arthrosc ‐ J Arthrosc Relat Surg. 2017;33:891–897. 10.1016/j.arthro.2016.09.029 28017469

[69] Provencher MT , Ghodadra N , LeClere L , Solomon DJ , Romeo AA . Anatomic osteochondral glenoid reconstruction for recurrent glenohumeral instability with glenoid deficiency using a distal tibia allograft. Arthrosc ‐ J Arthrosc Relat Surg. 2009;25:446–452. 10.1016/j.arthro.2008.10.017 19341934

[70] Rayes J , Xu J , Sparavalo S , Ma J , Jonah L , Wong I . Calculating glenoid bone loss based on glenoid height using ipsilateral three‐dimensional computed tomography. Knee Surg Sports Traumatol Arthrosc. 2023;31:169–176. 10.1007/s00167-022-07020-4 35674771

[71] Rohman E , Gronbeck K , Tompkins M , Mittelsteadt M , Kirkham JA , Arciero RA . Scapular spine dimensions and suitability as a glenoid bone graft donor site. Am J Sports Med. 2019;47:2469–2477. 10.1177/0363546519861965 31310727

[72] Rowe P , Koller A , Sharma S . Physiology, bone remodeling. In: StatPearls. Treasure Island (FL): StatPearls Publishing; 2025. 29763038

[73] Sayegh ET , Mascarenhas R , Chalmers PN , Cole BJ , Verma NN , Romeo AA . Allograft reconstruction for glenoid bone loss in glenohumeral instability: a systematic review. Arthrosc ‐ J Arthrosc Relat Surg. 2014;30:1642–1649. 10.1016/j.arthro.2014.05.007 24999006

[74] Shin S‐J , Kim RG , Jeon YS , Kwon TH . Critical value of anterior glenoid bone loss that leads to recurrent glenohumeral instability after arthroscopic bankart repair. Am J Sports Med. 2017;45:1975–1981. 10.1177/0363546517697963 28333542

[75] Singh M , Byrne R , Chang K , Nadella A , Kutschke M , Callanan T , et al. Distal tibial allograft for the treatment of anterior shoulder instability with glenoid bone loss: a systematic review and meta‐analysis. Am J Sports Med. 2025;53:210–216. 10.1177/03635465231223124 38384193

[76] Song Q , Zhang S , Cheng X , Xiao J , Lin L , Liu Q , et al. Clinical and radiographic outcomes after arthroscopic inlay bristow surgery with screw versus suture button fixation: a comparative study of 117 patients with 3.3‐year follow‐up. Orthop J Sports Med. 2022;10:232596712210760. 10.1177/23259671221076048 PMC8908399 35284584

[77] Tanaka M , Hanai H , Kotani Y , Kuratani K , Nakai H , Kinoshita S , et al. Open bristow versus open latarjet for anterior shoulder instability in rugby players: radiological and clinical outcomes. Orthop J Sports Med. 2022;10:23259671221095094. 10.1177/23259671221095094 PMC9118436 35601734

[78] Taverna E , Golanò P , Pascale V , Battistella F . An arthroscopic bone graft procedure for treating anterior‐inferior glenohumeral instability. Knee Surg Sports Traumatol Arthrosc. 2008;16:872–875. 10.1007/s00167-008-0541-y 18536907

[79] Taverna E , D'Ambrosi R , Perfetti C , Garavaglia G . Arthroscopic bone graft procedure for anterior inferior glenohumeral instability. Arthrosc Tech. 2014;3:e653–e660. 10.1016/j.eats.2014.08.002 PMC4314560 25685669

[80] Taverna E , Garavaglia G , Perfetti C , Ufenast H , Sconfienza LM , Guarrella V . An arthroscopic bone block procedure is effective in restoring stability, allowing return to sports in cases of glenohumeral instability with glenoid bone deficiency. Knee Surg Sports Traumatol Arthrosc. 2018;26:3780–3787. 10.1007/s00167-018-4921-7 29623353

[81] Tokish JM , Fitzpatrick K , Cook JB , Mallon WJ . Arthroscopic distal clavicular autograft for treating shoulder instability with glenoid bone loss. Arthrosc Tech. 2014;3:e475–e481. 10.1016/j.eats.2014.05.006 PMC4175166 25264509

[82] Tokish JM , Fitzpatrick K , Cook JB , Mallon WJ . Arthroscopic distal clavicular autograft for treating shoulder instability with glenoid bone loss. Arthrosc Tech. 2014;3:e475–e481. 10.1016/j.eats.2014.05.006 PMC4175166 25264509

[83] Tucker A , Ma J , Sparavalo S , Coady CM , Wong I . Arthroscopic anatomic glenoid reconstruction has a lower rate of recurrent instability compared to arthroscopic Bankart repair while otherwise maintaining a similar complication and safety profile. J ISAKOS. 2022:S2059775422000669. 10.1016/j.jisako.2022.05.003 35649503

[84] Verweij LPE , Schuit AA , Kerkhoffs GMMJ , Blankevoort L , van den Bekerom MPJ , van Deurzen DFP . Accuracy of currently available methods in quantifying anterior glenoid bone loss: controversy regarding gold standard—a systematic review. Arthrosc ‐ J Arthrosc Relat Surg. 2020;36:2295–2313.e1. 10.1016/j.arthro.2020.04.012 32330485

[85] Wang Y , Zhou Z , Zhang Y , He C , Xue C , Xu W , et al. Early follow‐up of arthroscopic latarjet procedure with screw or suture‐button fixation for recurrent anterior shoulder instability. Orthop Surg. 2020;12:1350–1361. 10.1111/os.12781 PMC7670134 33200576

[86] Weil S , Arnander M , Pearse Y , Tennent D . Reporting of glenoid bone loss measurement in clinical studies and the need for standardization: a systematic review. Bone Jt J. 2022;104–B:12–18. 10.1302/0301-620X.104B1.BJJ-2021-0751.R1 34969273

[87] Wong IH , King JP , Boyd G , Mitchell M , Coady C . Radiographic analysis of glenoid size and shape after arthroscopic coracoid autograft versus distal tibial allograft in the treatment of anterior shoulder instability. Am J Sports Med. 2018;46:2717–2724. 10.1177/0363546518789348 30095986

[88] Wong IH , Urquhart N . Arthroscopic anatomic glenoid reconstruction without subscapularis split. Arthrosc Tech. 2015;4:e449–e456. 10.1016/j.eats.2015.04.005 PMC4662065 26697303

[89] Wong I , John R , Ma J , Coady CM . Arthroscopic anatomic glenoid reconstruction using distal tibial allograft for recurrent anterior shoulder instability: clinical and radiographic outcomes. Am J Sports Med. 2020;48:3316–3321. 10.1177/0363546520960119 33044836

[90] Xiang M , Yang J , Chen H , Hu X , Zhang Q , Li Y , et al. Arthroscopic autologous scapular spine bone graft combined with bankart repair for anterior shoulder instability with subcritical (10%‐15%) glenoid bone loss. Arthrosc ‐ J Arthrosc Relat Surg. 2021;37:2065–2074. 10.1016/j.arthro.2021.01.061 33581303

[91] Yang JS , Mehran N , Mazzocca AD , Pearl ML , Chen VW , Arciero RA . Remplissage versus modified latarjet for off‐track hill‐sachs lesions with subcritical glenoid bone loss. Am J Sports Med. 2018;46:1885–1891. 10.1177/0363546518767850 29672132

[92] Zhao J , Huangfu X , Yang X , Xie G , Xu C . Arthroscopic glenoid bone grafting with nonrigid fixation for anterior shoulder instability: 52 patients with 2‐ to 5‐year follow‐up. Am J Sports Med. 2014;42:831–839. 10.1177/0363546513519227 24510068

[93] Zhu Y‐M , Jiang C‐Y , Lu Y , Li F‐L , Wu G . Coracoid bone graft resorption after Latarjet procedure is underestimated: a new classification system and a clinical review with computed tomography evaluation. J Shoulder Elbow Surg. 2015;24:1782–1788. 10.1016/j.jse.2015.05.039 26163284

